# Pulsed-Field Ablation for Atrial Premature Complexes From the Right Atrial Appendage Apex: A Novel Application

**DOI:** 10.1016/j.jaccas.2025.105903

**Published:** 2025-12-03

**Authors:** Lu Geng, Zhanxin Zhang, Yaqiong Jin, Jie Zhang, Jiaqi Wang, Li Wang, Keke Wang, Jingchao Lu

**Affiliations:** Department of Cardiology, The Second Hospital of Hebei Medical University, Shijiazhuang, Hebei Province, China

**Keywords:** atrial premature complexes, cardiac electrophysiology, catheter ablation, pulsed-field ablation, right atrial appendage

## Abstract

**Background:**

Atrial premature complexes (APCs) refractory to medical therapy pose management challenges. Pulsed-field ablation (PFA) is established for atrial fibrillation but is unreported for focal APCs.

**Case Summary:**

A 67-year-old woman with hypertension and hypothyroidism presented with 6-year refractory APCs (35.7% burden). Initial radiofrequency ablation at the right atrial appendage (RAA) apex failed owing to safety concerns. Subsequent PFA eliminated the APCs after 8 applications.

**Discussion:**

This first report demonstrates the efficacy of PFA for APCs originating from the RAA apex, avoiding the risks of thermal ablation in thin-walled structures.

**Take-Home Messages:**

PFA safely eliminates arrhythmias originating from the thin-walled RAA apex. This novel approach avoids thermal ablation risks in high-risk anatomical sites.

## Graphical Abstract

**Figure:**
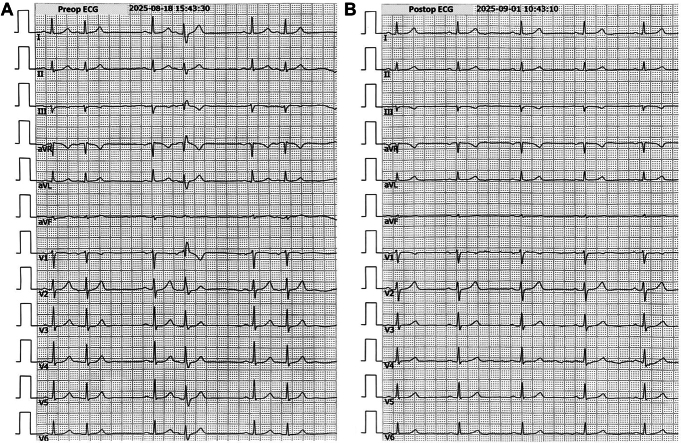

## History of Presentation

A 67-year-old woman was admitted to the hospital in August 2025 given intermittent palpitations over a 6-year period. Six years prior, she began experiencing palpitations accompanied by chest tightness and shortness of breath after physical activity. These episodes lasted several seconds and resolved with rest; she reported no associated chest pain, dizziness, or other symptoms. She was diagnosed with atrial premature complexes (APCs) at a local hospital and was prescribed antiarrhythmic medication, including propafenone. However, her palpitations showed no significant improvement, substantially affecting her quality of life. On admission, her heart rate was 55 beats/min with an irregular rhythm and audible premature beats; her blood pressure was 110/75 mm Hg. Physical examination was otherwise unremarkable.

**Take-Home Messages**

• PFA safely eliminates arrhythmias originating from the thin-walled right atrial appendage apex.
• This novel approach avoids the risks of thermal ablation in high-risk anatomical sites.

## Past Medical History

The patient's medical history included hypertension for 5 years and hypothyroidism for 1 year. Both conditions were well controlled with medication, with blood pressure and thyroid function within normal ranges. She had no prior cardiac interventions or device implantations.

## Differential Diagnosis

The differential diagnoses included atrial tachycardia, sinoatrial nodal re-entrant tachycardia, and focal atrial fibrillation. These were excluded by electrophysiological study demonstrating isolated APCs without sustained arrhythmia.

## Investigations

**Figure 1:**
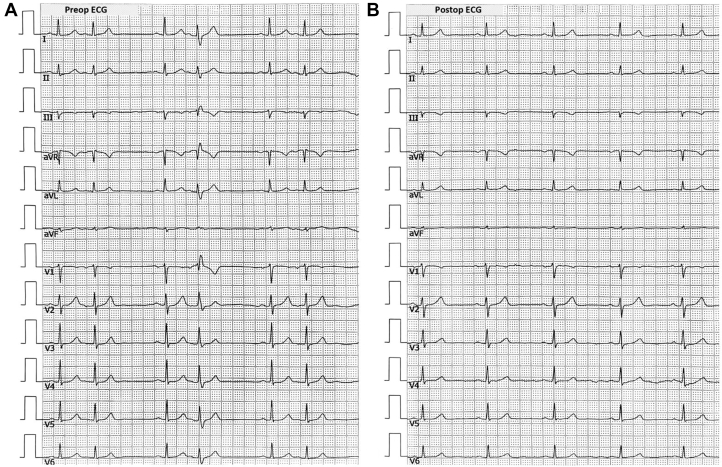
Preprocedural and Postprocedural Electrocardiogram Tracings (A) Preprocedural electrocardiogram demonstrating atrial bigeminy. (B) Postprocedural electrocardiogram confirming resolution of atrial premature complexes.

**Figure 2:**
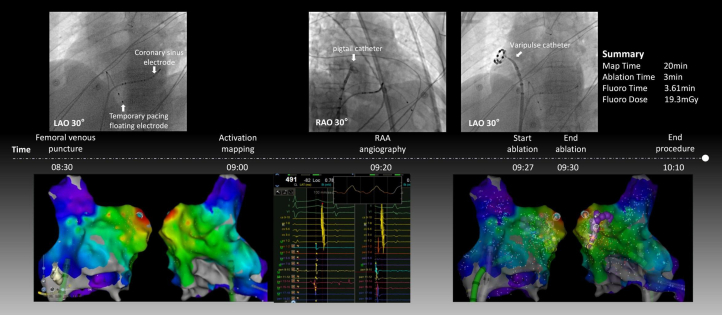
Procedural Workflow for Pulsed-Field Ablation of Atrial Premature Complexes Originating From the Right Atrial Appendage Procedural workflow: Performed under general anesthesia with pacing backup. Activation mapping used a PentaRay catheter advanced through a Vizigo sheath. Right atrial appendage anatomy was defined by angiography. PFA was delivered via Varipulse catheter under Carto 3 guidance. Fluoroscopy time/dose: 3.61 min/19.3 mGy. LAO = left anterior oblique; PFA = pulsed-field ablation; RAA = right atrial appendage; RAO = right anterior oblique.

A 12-lead electrocardiogram confirmed atrial bigeminy (Figure 1A), providing immediate electrophysiological evidence for the symptomatic presentation. Results of 24-hour Holter monitoring performed 2 weeks prior revealed significant arrhythmia burden: among 90,398 total heartbeats, 31,314 were APCs (35.7% burden), including 555 episodes of atrial bigeminy, quantitatively explaining the persistent palpitations. Transthoracic echocardiography demonstrated enlargement of the right atrium (41 mm), right ventricle (41 mm), and left atrium (40 mm), accompanied by moderate tricuspid regurgitation (peak regurgitant velocity: 291 cm/s) and elevated pulmonary artery systolic pressure (42 mm Hg), whereas left ventricular ejection fraction remained preserved at 67.9%. Routine laboratory investigations including complete blood count, thyroid function, and cardiac biomarkers all fell within normal limits. Three-dimensional mapping localized the earliest activation site at the right atrial appendage (RAA) apex, characterized by a distinctive double potential preceding the surface P-wave. The procedural workflow detailing catheter positioning and energy delivery is comprehensively illustrated in Figure 2.

## Management

After comprehensive electrophysiological mapping confirmed the RAA apex as the arrhythmia origin, conventional radiofrequency catheter ablation (RFCA) was initially attempted. Using a contact-force sensing catheter (SmartTouch), ablation was performed at the target site with temperature-controlled settings (43 °C, 30 W) for 90 seconds under stable contact force (4*g*-5*g*). Despite optimal catheter positioning and energy delivery, the APCs persisted intraprocedurally. Given the risk of RAA perforation and cardiac tamponade with further ablation attempts at this location, the procedure was terminated.

After multidisciplinary consultation, pulsed-field ablation (PFA) was performed under general anesthesia with temporary pacing support. Activation mapping was conducted using a PentaRay catheter advanced through a Vizigo sheath, which identified the earliest activation site at the RAA apex. Angiography was performed via a pigtail catheter to delineate RAA anatomy. Under Carto 3 guidance, a Varipulse catheter was positioned; it incorporated tissue proximity indication technology, which detected impedance changes exceeding 7% to provide real-time three-dimensional visualization of catheter-tissue contact, enabling precise intervention at the target site. After the catheter was introduced into the cardiac chamber, the loop diameter was adjusted to the minimum setting of 25 mm. Because the loop was constructed from temperature-sensitive alloy, its actual diameter contracted to 15 to 18 mm within the human body, facilitating advancement into the apical region of the atrial appendage for accurate targeting. During the procedure, once the catheter was positioned near the appendage apex, electrodes 1, 2, and 10 were deactivated given their close proximity resulting from the reduced loop size. The catheter position was then fine-tuned until tissue proximity indication confirmed optimal contact with all 7 remaining electrodes against the appendage wall. Energy delivery was subsequently initiated under these conditions to complete the ablation.

Initial PFA applications at the free wall of the RAA apex failed to eliminate all APCs; however, subsequent ablation at the septal aspect completely abolished the arrhythmia, with a total of 8 PFA applications delivered. Postprocedural electrophysiological mapping demonstrated decremental conduction, with a Wenckebach cycle length of 350 ms during S_1_S_1_ pacing from the CS_7-8_ electrodes. Ventricular noncapture was observed during S_1_S_2_ stimulation (500/270 ms) (Figure 2, Table 1). The procedure was ultimately successful, with a postprocedural electrocardiogram confirming sinus rhythm (Figure 1B). A video summary of the procedure is available as a supplement (Video 1).

**Table 1:** Equipment List

Category and Device	Manufacturer	Key Application
Mapping and navigation		
Carto 3 three-dimensional mapping system	Biosense Webster	Electroanatomic modeling and catheter navigation
PentaRay high-density mapping catheter	Biosense Webster	Localization of earliest activation at RAA apex
Vizigo steerable sheath	Biosense Webster	Precise catheter delivery to RAA apex
Ablation		
Varipulse pulsed-field ablation catheter	Biosense Webster	Nonthermal ablation avoiding thin-wall perforation
Auxiliary		
Pigtail catheter	Cordis	RAA angiography (anatomic definition)
Temporary pacemaker	Medtronic	Intraprocedural pacing support

RAA = right atrial appendage.

## Outcome and Follow-Up

The patient reported complete resolution of palpitations at the 1-week follow-up. Repeat 24-hour Holter monitoring demonstrated 312 APCs (<1% burden vs 35.7% before the procedure). Antiarrhythmic medications were successfully discontinued without recurrence of symptoms. This outcome confirms the durable efficacy of PFA for eliminating arrhythmogenic foci in high-risk anatomical sites.

## Discussion

The management of arrhythmias originating from the RAA demands meticulous consideration of its unique anatomical and electrophysiological properties. The RAA's thin-walled, trabeculated structure, characterized by pectinate muscles and variable morphology (eg, saccular, multilobulated, or anomalous pouches), poses significant challenges for catheter stability and transmural lesion formation. Conventional RFCA often fails in distal RAA regions owing to inadequate tissue contact, limited power delivery (to avoid perforation), and heat dissipation in low–blood flow environments. Furushima et al^1^ exemplified these limitations, where RFCA at the RAA apex failed despite 50 applications, ultimately necessitating surgical appendectomy. This highlights the anatomical constraints of RFCA, particularly in regions with complex trabeculae or dead-end cavities.

Cryoballoon ablation has emerged as an alternative for proximal RAA foci, leveraging its ability to create broader, adherent lesions. Amasyali et al^2^ and Yorgun et al^3^ achieved acute success using balloon occlusion at the RAA base. However, cryoballoons face limitations in distal or narrow RAA apices where complete occlusion is unfeasible, risking incomplete lesion formation. For such refractory cases, minimally invasive thoracoscopic clamp ablation offers direct epicardial access, especially valuable in pediatric patients or tachycardia-induced cardiomyopathy.^4^ Yet its invasiveness, requirement of surgical expertise, and potential for atrial mechanical dysfunction underscore the need for less traumatic alternatives. Technical innovations such as contrast-guided irrigation catheters improved mapping precision in distal RAA regions but added procedural complexity.^5^

The advent of PFA has revolutionized RAA management by addressing the core limitations of thermal ablation. PFA's nonthermal, cardiomyocyte-selective mechanism, which uses high-voltage microsecond pulses to electroporate cell membranes, enables efficient lesion creation without collateral damage to phrenic nerves or esophageal structures. Urbanek et al^6^ first demonstrated the efficacy of PFA in incessant RAA tachycardia, where 8 applications terminated arrhythmia without complications. Crucially, PFA's efficacy persists in anomalous RAA anatomy,^7^ where traditional RFCA struggles owing to impedance variability and unstable catheter contact. Rattanawong et al.^8^ expanded this use to RAA isolation in the management of atrial fibrillation, achieving acute success with focal flower/basket configurations while preserving sinus node function (Table 2).

**Table 2:** Studies Reporting Management Strategies for Arrhythmias of RAA Origin

Lead Author (Year)	Sex	Age	Arrhythmia Type	Origin Location	Prior Procedures	Prior Device	Current Procedure	Current Device	Follow-Up Outcomes
Furushima (2009)^1^	F	44	Focal AT (90% burden)	RAA apex	2 failed RFCA (50 lesions, 58 min)	2 4-mm-tip ablation catheters (NaviStar and Celsius); an 8-mm-tip catheter (Ablase)	Surgical forceps	Surgical resection	9 mo recurrence-free
Amasyali (2015)^2^	M	48	Focal AT	RAA base and midportion	1 failed RFCA (43 °C, 0-35 W)	NaviStar ThermoCool quadripolar 4-mm cooled-tip navigation catheter	Cryoablation (−43 °C, 240 s, −45 °C, 240 s)	Arctic Front 28-mm cryoballoon; FlexCath 12-F steerable introducer; Achieve circular mapping catheter	LVEF 61% at 6 mo (prior 40%)
Yorgun (2019)^3^	M	26	Focal AT	Anterosuperior RAA	1 failed RFCA	NaviStar ThermoCool 3.5-mm irrigated tip catheter	Cryoablation (−47 °C, 240 s, −48 °C, 300 s)	Arctic Front 28-mm cryoballoon; Achieve circular mapping catheter	LVEF 45% at 1 mo (prior 25%)
Luo (2021)^4^	M	13	Focal AT	RAA apex	1 failed RFCA (50 lesions, 50 min; 15-25 W, 43 °C)	PentaRay mapping catheter; ThermoCool ablation catheter	Thoracoscopic clamp ablation (3 times, 28.5 W)	Medtronic bipolar RF clamp	LVEF 60% at 3 mo (prior 31%)
Nishizaki (2021)^5^	F	30	Focal AT	Posterolateral RAA apex	Prior failed ablation	Conventional mapping/ablation catheter	Contrast-guided RFCA (41 °C, 15 W)	IntellaNav MiFi Ol steerable catheter; Rhythmia system; IntellaMap Orion multielectrode catheter	LVEF 53% at 12 mo (prior 29%)
Urbanek (2022)^6^	—	36	Focal AT	Broad zone at RAA base	2 failed RFCA	Standard RF ablation catheter (model not reported)	PFA (8 times, 20 s)	Farawave catheter (31 mm, flower/basket shape); PentaRay mapping catheter	Acute success, no recurrence
Amin (2024)^7^	M	58	Focal AT	Anomalous RAA lobe (SVC-originated accessory pouch)	Failed DCCV ×3; first ablation: AT slowed but recurred (40-50 W)	PentaRay mapping catheter; ThermoCool SmartTouch RF catheter	ICE-guided RFCA (50 W, 10-20 g)	ICE; ThermoCool SmartTouch RF catheter	LVEF 45% at 4 mo (prior 10%)
Rattanawong (2025)^8^	(1) M (2) M	(1) 69 (2) 53	(1) Symptomatic paroxysmal AF (2) Symptomatic persistent AF	(1 and 2) RAA-SVC junction (APCs initiating AF)	(1) — (2) Failed PVI (11 y prior)	(1) — (2) Standard RF ablation catheter (model not reported)	(1) PFA-guided RAA isolation (10 times) (2) PFA-guided RAA isolation (8 times)	(1 and 2) ICE; Farawave catheter (flower/basket configurations)	(1) 10 mo AF-free (2) 9 mo AF-free
Our Case (2025)	F	67	APCs (35.7% burden)	RAA apex	1 failed RFCA (43 °C, 30 W, 90 s, 4*g*-5*g* contact force)	ThermoCool SmartTouch RF catheter	PFA (8 times, 3 min)	Varipulse catheter (25 mm, circular array shape); Vizigo steerable sheath; PentaRay mapping catheter	<1% APC burden at 1 wk (prior 35.7%)

APCs = atrial premature complexes; AF = atrial fibrillation; AT = atrial tachycardia; DCCV = direct current cardioversion; F = female; ICE = intracardiac echocardiography; LVEF = left ventricular ejection fraction; M = male; PFA = pulsed-field ablation; PVI = pulmonary vein isolation; RAA = right atrial appendage; RF = radiofrequency; RFCA = radiofrequency catheter ablation; SVC = superior vena cava.

Although prior studies demonstrated PFA efficacy using the Farawave catheter,^6^^,^^8^ our case highlights the critical advantages of the Varipulse system in this challenging anatomical context. Unlike the rigid 31-mm Farawave flower/basket design, which risks incomplete contact in multilobulated RAA apices, the variable-loop circular array of the Varipulse enables precise navigation within narrow trabeculated spaces, as confirmed by real-time Carto 3 integration with contrast-enhanced anatomy merging. This allows targeted energy delivery from both the free wall and the septal aspect without requiring complete anatomical occlusion—a limitation noted in Farawave applications near the superior vena cava junction. Furthermore, Varipulse's programmable biphasic waveform significantly reduces phrenic nerve stimulation risk during apical ablation compared with Farawave's fixed-output pulses, while achieving acute arrhythmia termination with fewer applications.^8^ For anomalous RAA pouches, where Amin et al^7^ documented RFCA failure due to unstable contact, Varipulse's adaptive design and steerable sheath (Vizigo) provide superior stability. These technical refinements position Varipulse as the optimal PFA platform for RAA apex arrhythmias, overcoming Farawave's anatomical constraints while enhancing safety and efficiency.

## Funding Support and Author Disclosures

The authors have reported that they have no relationships relevant to the contents of this paper to disclose.

## Appendix

Video 1 Summary of the Pulsed-Field Ablation Procedure for Atrial Premature Complexes at the Right Atrial Appendage Apex
